# Mortality of Adult Patients With Cancer Admitted to an Intensive Care Unit in Chile: A Prospective Cohort Study

**DOI:** 10.1200/JGO.18.00091

**Published:** 2018-11-30

**Authors:** Sergio Panay, Carolina Ruiz, Marcelo Abarca, Bruno Nervi, Ignacio Salazar, Paulo Caro, Sabrina Muñiz, Juan Briones, Alejandro Bruhn, Sebastian Mondaca

**Affiliations:** **Sergio Panay**, **Carolina Ruiz**, **Marcelo Abarca**, **Ignacio Salazar**, **Paulo Caro**, **Sabrina Muñiz**, and **Juan Briones**, Complejo Asistencial Dr. Sótero del Río; **Carolina Ruiz** and **Alejandro Bruhn**, Universidad Católica de Chile; and **Carolina Ruiz, Alejandro Bruhn**, **Bruno Nervi**, and **Sebastian Mondaca**, Pontificia Universidad Católica de Chile, Santiago, Chile.

## Abstract

**Purpose:**

Increasing numbers of reports have shown acceptable short-term mortality of patients with cancer admitted into the intensive care unit (ICU). The aim of this study was to determine the mortality of critically ill patients with cancer admitted to the ICU in a general hospital in Chile.

**Materials and Methods:**

This was a prospective cohort trial in which we included all patients with cancer admitted to the ICU between July 2015 and September 2016. Demographic, physiologic, and treatment data were registered, and survival at 30 days and 6 months was evaluated. A prespecified subgroup analysis considering the admission policy was performed. These subgroups were (1) ICU admission for full code management and (2) ICU trial (IT).

**Results:**

During the study period, 109 patients with cancer were included. Seventy-nine patients were considered in the full code management group and 30 in the IT. The mean age of patients was 60 years (standard deviation [SD], 15), and 56% were male. Lymphoma was the most frequent malignancy (17%), and 59% had not received cancer treatment because of a recent diagnosis. The mean Acute Physiology and Chronic Health Evaluation and Sequential-Related Organ Failure Assessment scores were 22.2 (SD, 7.3) and 7 (SD, 3), respectively. There were no differences in vasopressor, fluid, or transfusion requirements between subgroups. Lactate levels, Sequential-Related Organ Failure Assessment scores (day 1, 3, and 5), complications, and ICU length of stay were similar. In the entire cohort, 30-day and 6-month mortality was 47% and 66%, respectively. There was no difference in mortality between subgroups according to the admission policy.

**Conclusion:**

Patients admitted to the ICU in a developing country are at high risk for short-term mortality. However, there is a relevant subgroup that achieves 6-month survival, even among patients who undergo an IT.

## INTRODUCTION

Over the past decades, the prognosis of many malignancies has improved significantly. This can be partially explained by major advances in both early cancer detection and the development of new, more effective treatments.^1^ Indeed, the development of targeted therapy and more recently immunotherapy have made a positive impact on outcomes of patients with advanced disease.^2^ Concomitantly, in recent years we have also witnessed a transition in the approach to cancer care regarding patients’ admission to the intensive care unit (ICU). Thirty years ago, the ICU admission policies were restrictive, given the bad short- and medium-term prognoses observed in these patients.^3^ However, many recent experiences, mainly derived from academic centers, have shown better outcomes in patients with cancer, probably due to improvements in ICU management and cancer care.^4-6^ These outcomes are currently comparable to those observed in patients with other chronic conditions, such as heart failure or liver cirrhosis.^7^ To identify the patients who will benefit from these interventions, several prognostic factors have been proposed, including the need for vasopressors and for invasive mechanical ventilation^8,9^; however, their use in clinical practice is limited mainly because of their lack of external validation and limited predictive power in the context of new treatments.^6^

In 2007, a French study reported an admission policy called the ICU trial (IT) that aimed to identify patients with cancer who could benefit from a limited time of active treatment in the ICU. This study demonstrated that organ dysfunction at day 6 was more effective at predicting patient survival compared with organ dysfunction at the time of admission.^10^ This strategy has been incorporated into patient care guidelines and is generally accepted by the international community,^11,12^ but some aspects, such as the optimal duration of IT, are still under research.^13^ In developing countries, the data supporting the effectiveness of IT are scarce. Multiple variables could affect the applicability of IT in limited-resource settings. In these countries, the care in the ICU and the access to standard oncologic treatments might be suboptimal; this could lead to poor outcomes, affecting the rationale of admitting patients with cancer to the ICU. Moreover, ICU admission in this setting is associated with lower satisfaction levels among family members regarding end-of-life care of patients.^14^ Hence, the aim of our study was to evaluate mortality rates in a cohort of patients with cancer at 30 days and 6 months after their admission into the ICU at a Chilean public hospital with limited resources. A prespecified subgroup analysis considering the admission policy was included, comparing patients who underwent an IT and the full code management (FCM) subgroup.

## MATERIALS AND METHODS

This prospective cohort trial was conducted at the Complejo Asistencial Dr. Sótero del Río (CASR), located in Santiago, Chile. This is a high-volume public hospital and is responsible for the oncologic care of 1.6 million people. CASR has 78 intensive care beds; in 18 of them, it is possible to give invasive ventilatory support, and in the other 60 beds, noninvasive mechanical ventilation is available.

### Patients

We included patients with cancer older than 18 years of age, with baseline Eastern Cooperative Oncology Group classification from 0 to 3, who were admitted to the ICU because of one of the following criteria: (1) indication of invasive or noninvasive ventilatory support because of acute respiratory failure, (2) use of vasopressor drugs because of hypotension, or (3) renal replacement therapy in the context of acute kidney injury. Patients were recruited between July 2015 and September 2016. Respiratory failure was defined as pulse oximetry less than 90% or a partial pressure of arterial oxygen less than 60 mm Hg with a fraction of inspired oxygen greater than 50%. Use of vasopressor drugs because of hypotension was defined as administration of noradrenaline greater than 0.1 µg/kg/min for persistent hypotension (mean arterial pressure less than 65 mm Hg). The indication for renal replacement therapy was defined by the intensivist, but institutional recommendation is based on KDIGO (Kidney Disease: Improving Global Outcomes) guidelines.^15^ Patients were required to have histologically confirmed cancer, and if the diagnosis of cancer was based on clinical assessment, it had to be histologically confirmed during the admission. Patients without recurrence of cancer in 5 years or who had nonmelanoma skin cancer were excluded.

### Data Collection

Demographic, physiologic, laboratory, and treatment data were extracted from medical records. The hospital length of stay, 30-day and 6-month mortality, and cancer treatment (chemotherapy and radiotherapy) were also registered. Mortality rates were assessed using the hospital and national mortality registry database. A prespecified subgroup analysis was performed considering the admission policy. As part of routine care, when a patient with cancer was presented for ICU admission, an intensivist evaluated the patient and defined one of the following therapeutic plans: (1) palliative care for patients with a bad performance status and/or a poor oncologic prognosis who were not admitted to the ICU because it was not considered to be beneficial; (2) ICU admission for FCM for patients who were candidates for receiving active cancer treatment with a reasonable chance of disease control; in this group, unlimited interventions were considered similarly to critically ill patients without cancer; or (3) IT for patients with intermediate prognosis who did not meet the criteria for inclusion in the previous two groups and who could benefit from a limited time of advanced interventions. For patients considered for palliative care, the 30-day and 6-month mortality was also determined. The study team was not involved in the decision of the therapeutic plan nor in any other treatment decision.

### Statistical Analysis

The primary end point was 30-day mortality, and we included 6-month mortality as a medium-term outcome following recommendations for trial design in critically ill patients.^16^ As secondary end points, we assessed 30-day and 6-month mortality for the specific subgroups of ICU therapeutic plans. A sample size of 90 patients was estimated for this study to determine the main outcome to within +/- 10% margin of error with 95% confidence. The characteristics of this cohort were expressed as median and interquartile range (IQR) or mean and standard deviation (SD) for continuous variables and as percentages for categorical variables. We used the Mann-Whitney test for the analyses of quantitative variables and the χ^2^ test for qualitative variables. A two-tailed *P* value of .05 was considered statistically significant. We performed a multivariable analysis using a logistic regression to estimate the independent association of each variable with 30-day mortality. The variables that had a *P* value < .2 in the univariable analysis were considered in the multivariable analysis. The results were reported as odds ratio and its 95% CI. Statistical analyses were performed using the SPSS software, version 21 (SPSS, Chicago, IL). The ethics committee of CASR approved the study and allowed the research team to dispense with consent because of the observational nature of the trial. This trial is registered at ClinicalTrials.gov (NCT02659839).

## RESULTS

### Patients

**Fig 1:**
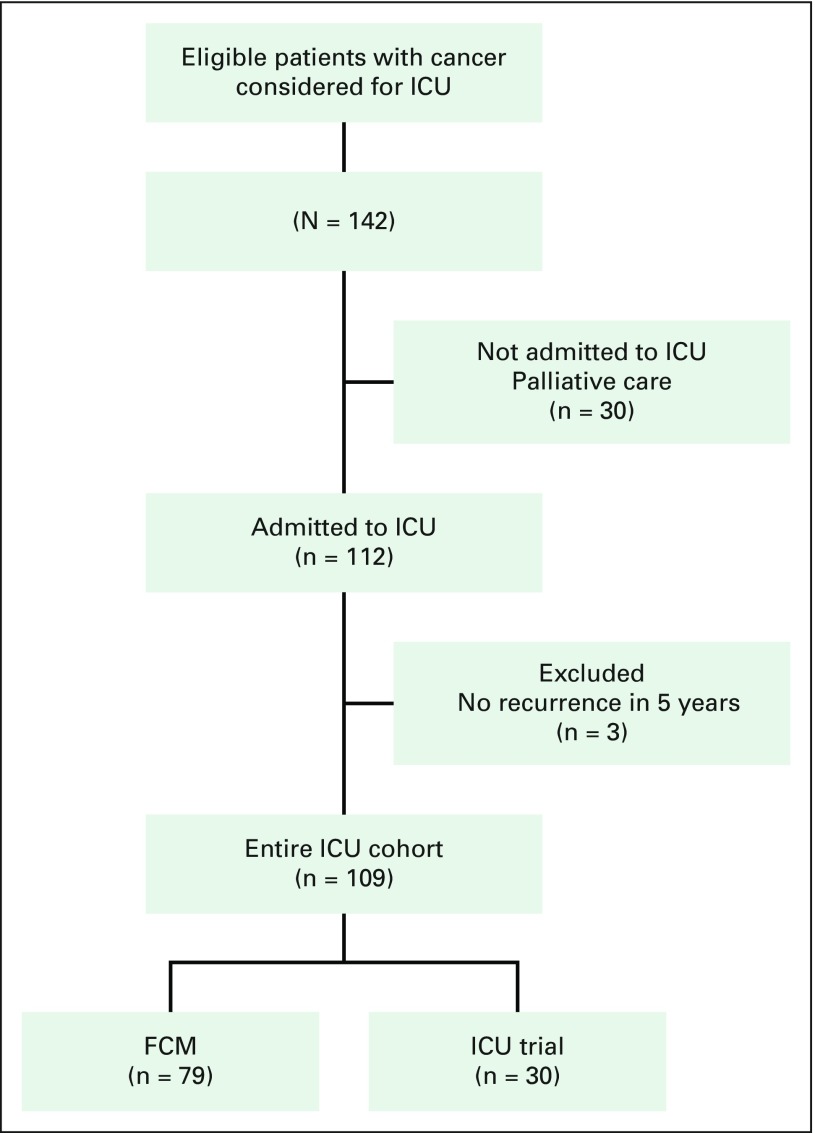
Flow diagram of the study. ICU, intensive care unit; FCM, full code management.

A total of 3,589 patients were admitted to the CASR ICU between July 2015 and September 2016. A total of 142 patients with cancer were evaluated for admission into the ICU in this period because of at least one of the three main inclusion criteria (acute respiratory failure, hypotension despite fluid resuscitation, or acute kidney injury). Thirty patients were not admitted to the ICU and sorted into the palliative care group. Of the patients admitted to the ICU, three did not meet the inclusion criteria. Therefore, 109 patients were included in the final analysis of patients admitted to the ICU. Within this group, 79 patients were in the FCM subgroup, and 30 were in the IT subgroup (Fig 1). Baseline characteristics of the population are listed in Table 1. The majority of patients (56%) were men, and their mean age was 60 years (range, 20 to 83 years). Fifty-five percent of patients had solid tumors, and the most frequent malignancy was lymphoma (17%). Regarding cancer status, 59% of patients had not received any therapy because of recent diagnosis, whereas 15% and 26% were in remission and progression, respectively. The most frequent criterion for ICU admission was respiratory failure (54%), followed by hemodynamic instability (39%). The Acute Physiology and Chronic Health Evaluation score was 22.2 (SD, 7.3), and the first-day Sequential-Related Organ Failure Assessment score was 7 (SD, 3). For the entire group, the median length of hospital stay was 27 days (IQR, 14 to 49 days), with a median of 8 days (IQR, 3 to 14 days) in the ICU. Patients in the FCM subgroup had a greater hospital length of stay (36 days; IQR, 17 to 52 days) than patients in the IT subgroup (16 days; IQR, 6 to 28 days; *P* = .004), whereas there was no difference in length of stay in the ICU (*P* = .09).

**Table 1:**
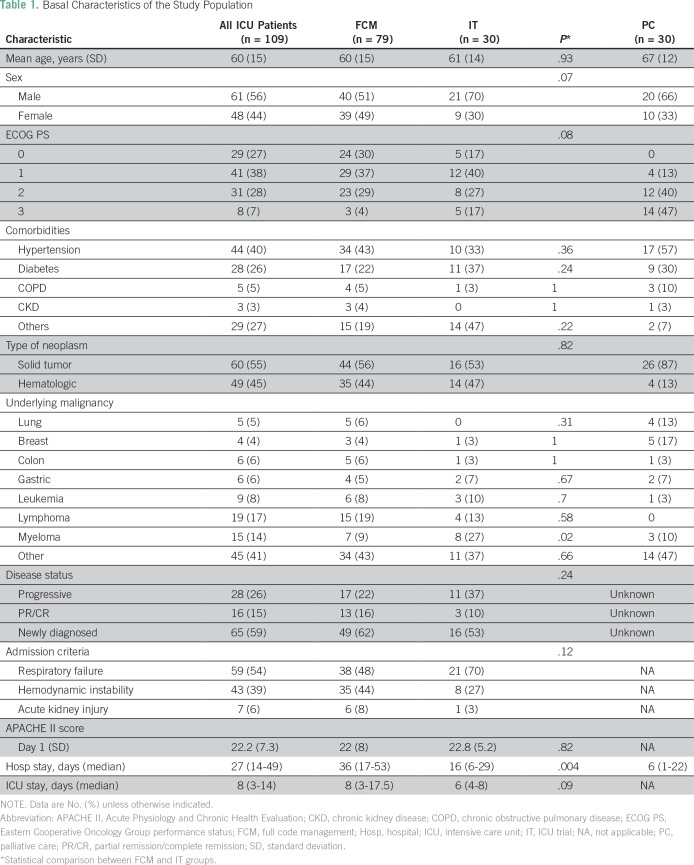
Basal Characteristics of the Study Population

### Treatments

Patients spent a median of 3 days (IQR, 1 to 13 days) in the emergency unit or other hospital services between hospital admission and ICU admission. Most patients (68%) needed vasoactive drugs during their stay in the ICU. The most frequently used vasoactive drug was noradrenaline, with a mean dose during the first day of 0.21 mcg/kg/min (SD, 0.19 mcg/kg/min). The median lactate level on ICU admission was 18 mEq/L (IQR, 11.4 to 44.6 mEq/L). During the ICU stay, 70% of patients required blood transfusions, and 13% required renal replacement therapy. None of these variables displayed significant differences between the FCM and IT subgroups (Table 2). Mechanical ventilatory support was used in 82% of patients (invasive, noninvasive, or both), and invasive ventilation was more frequent in the FCM subgroup compared with the IT subgroup (70% *v* 37%; *P* = .001; Table 2). Organ dysfunction evaluated through Sequential-Related Organ Failure Assessment scores was similar on days 1, 3, and 5 between subgroups.

**Table 2:**
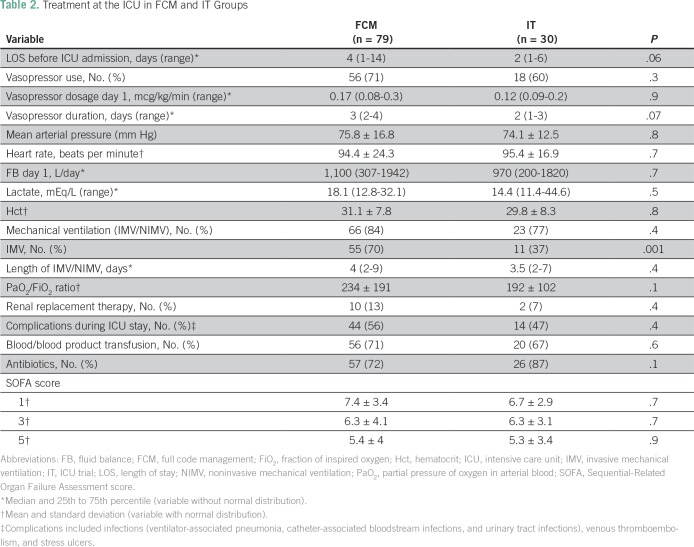
Treatment at the ICU in FCM and IT Groups

### Mortality

In the entire cohort, the 30-day mortality was 48%, and the 6-month mortality was 66%. In the FCM and IT subgroups, the mortality rates at 30 days were 43% and 60% (*P* = .11) and at 6 months were 67% and 63% (*P* = .71), respectively. No statistically significant differences were observed between subgroups (Table 3). After hospital discharge, 19 patients (17%) received systemic cancer treatment, six in the IT subgroup and 13 in the FCM subgroup. The mortality in patients who were not admitted to the ICU was 90% at 30 days and 97% at 6 months.

**Table 3:**
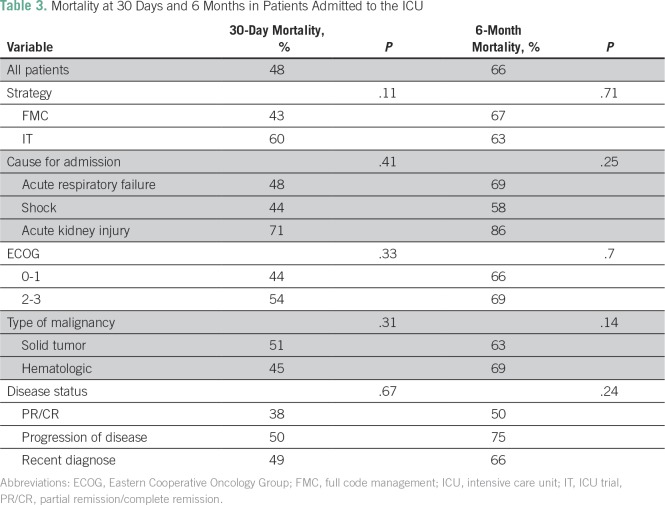
Mortality at 30 Days and 6 Months in Patients Admitted to the ICU

### Univariable and Multivariable Analysis

In the univariable analysis, higher lactate levels were associated with higher 30-day mortality, whereas invasive mechanical ventilation was associated with a lower 30-day mortality. In the multivariable analysis, no variable was found to be a predictor of 30-day mortality at the prespecified level of significance (Table 4).

**Table 4:**
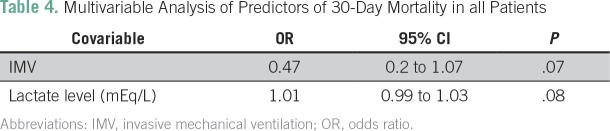
Multivariable Analysis of Predictors of 30-Day Mortality in all Patients

## DISCUSSION

In this observational study, we showed that in patients with cancer admitted to the ICU in a public hospital in Chile, the 30-day mortality and 6-month mortality were 48% and 66%, respectively. We also showed a trend toward higher 30-day mortality in the group treated in the context of an IT compared with the FCM group, but this difference disappeared at the 6-month analysis. These results seem poor compared with a retrospective review of unselected patients in the same ICU that reported a hospital mortality rate of 31%.^17^ Two studies conducted in Brazil and China found that the hospital mortality rates in patients with cancer admitted to the ICU were 30% and 29.8%, respectively.^18,19^ These differences could be explained by several factors. In particular, we speculate that selection of patients could be a key variable. In the first above-mentioned trial, 57% of patients were admitted for postoperative care, whereas our cohort only considered patients with specific severity criteria. In the retrospective analysis performed by Xia and Wang,^19^ the ICU mortality of all patients admitted during the study period was 4.3%, which suggests, to some extent, a lower-risk population. An additional factor that could explain the high mortality in our cohort is the time length between hospital and ICU admission. This might be related to reluctance to admit patients with cancer to the ICU or limited availability of ICU beds. An increasing amount of evidence indicates that early ICU admission of these patients leads to better outcomes.^20,21^ To the best of our knowledge, this is the first prospective study assessing the outcomes of the IT admission policy in a developing country. Interestingly, the original study that proposed this policy reported a hospital mortality rate of 78%,^10^ which is similar to our 30-day mortality in this subgroup.

This study has some limitations. First, it was conducted in a single center in Chile, which limits the applicability of some of our findings and highlights the need for further validation of these results in other cohorts in settings with limited resources. Second, we did not evaluate quality of life of patients, which has been recognized as an increasingly relevant outcome for patients with cancer.^22^ Third, the number of patients who participated in an IT is relatively small, which limits the precision of the estimates and precludes definitive conclusions. Among the strengths of our study, it was prospectively designed and is a real-world effectiveness analysis in a resource-limited setting. There are several future challenges and unknowns in the treatment of patients with cancer admitted to the ICU.^23^ To determine long-term outcomes and a tailored approach for the treatment of respiratory failure in these patients seems particularly relevant. From the perspective of a developing country, we would stress the importance of knowing the impact of subsequent treatments on the overall prognosis. In countries with limited resources, the access to state-of-the-art treatments is not universal,^24,25^ and for frail patients recovering from a long admission, access could be even more limited. A thorough cost-effective analysis is paramount to establish a policy in countries with many unmet needs in terms of health care.^26^

In conclusion, our cohort study shows that the mortality of patients with cancer admitted to the ICU is high, but there is a relevant subgroup that achieves a 6-month survival. In patients who participated in an IT, there was initially a trend toward higher mortality compared with full code patients, but at 6 months, we found no difference. Our data suggest that it is appropriate to admit patients with cancer to the ICU in developing countries considering these middle-term outcomes. More data are needed to confirm the cost-effectiveness of this strategy.
